# Induction Chemotherapy for Primarily Unresectable Locally Advanced Pancreatic Adenocarcinoma—Who Will Benefit from a Secondary Resection?

**DOI:** 10.3390/medicina57010077

**Published:** 2021-01-18

**Authors:** Nathalie Rosumeck, Lea Timmermann, Fritz Klein, Marcus Bahra, Sebastian Stintzig, Thomas Malinka, Uwe Pelzer

**Affiliations:** 1 Department of Hematology/Oncology/Tumorimmunology, Charité–Universitätsmedizin Berlin, Freie Universität Berlin, Humboldt-Universität zu Berlin and Institute of Health, 10117 Berlin, Germany; sebastian.stintzig@charite.de (S.S.); uwe.pelzer@charite.de (U.P.); 2 Department of Surgery, Charité–Universitätsmedizin Berlin, Freie Universität Berlin, Humboldt-Universität zu Berlin and Institute of Health, 10117 Berlin, Germany; lea.timmermann@charite.de (L.T.); fritz.klein@charite.de (F.K.); marcus.bahra@charite.de (M.B.); Thomas.Malinka@charite.de (T.M.)

**Keywords:** pancreatic cancer, neoadjuvant therapy, induction therapy, secondary resection, pancreatic surgery

## Abstract

*Background and Objectives:* An increasing number of patients (pts) with locally advanced pancreatic cancer (LAPC) are treated with an intensive neoadjuvant therapy to obtain a secondary curative resection. Only a certain number of patients benefit from this intention. The aim of this investigation was to identify prognostic factors which may predict a benefit for secondary resection. *Materials and Methods:* Survival time and clinicopathological data of pts with pancreatic cancer were prospective and consecutively collected in our Comprehensive Cancer Center Database. For this investigation, we screened for pts with primarily unresectable pancreatic cancer who underwent a secondary resection after receiving induction therapy in the time between March 2017 and May 2019. *Results:* 40 pts had a sufficient database to carry out a reliable analysis. The carbohydrate-antigen 19-9 (CA 19-9) level of the pts treated with induction therapy decreased by 44.7% from 4358.3 U/mL to 138.5 U/mL (*p* = 0.001). The local cancer extension was significantly reduced (*p* < 0.001), and the Eastern Cooperative Oncology Group (ECOG) performance status was lowered (*p* = 0.03). The median overall survival (mOS) was 20 months (95% CI: 17.2–22.9). Pts who showed a normal CA 19-9 level (<37 U/mL) at diagnosis and after neoadjuvant therapy or had a Body Mass Index (BMI) below 25 kg/m^2^ after chemotherapy had a significant prolonged overall survival (29 vs. 19 months, *p* = 0.02; 26 vs. 18 months, *p* = 0.04; 15 vs. 24 months, *p* = 0.01). Pts who still presented elevated CA 19-9 levels >400 U/mL after induction therapy did not profit from a secondary resection (24 vs. 7 months, *p* < 0.001). Nodal negativity as well as the performance of an adjuvant therapy lead to better mOS (25 vs. 15 months, *p* = 0.003; 10 vs. 25 months, *p* < 0.001). *Conclusion:* The pts in our investigation had different benefits from the multimodal treatment. We identified the CA 19-9 level at time of diagnosis and after neoadjuvant therapy as well as the preoperative BMI as predictive factors for overall survival. Furthermore, diagnostics of presurgical nodal status should gain more importance as nodal negativity is associated with better outcome.

## 1. Introduction

Pancreatic ductal adenocarcinoma (PDAC) is one of the most aggressive malignancies and a leading cause of cancer death worldwide. It is expected to become the second leading cause of cancer-related death within this decade [1,2]. The lack of effective targeted agents as well as missing validated predictive biomarkers that can probably facilitate therapeutic decision-making are major barriers in the treatment of pancreatic cancer.

In patients (pts) with inoperable disease and better performance status, two chemotherapy options, FOLFIRINOX (fluorouracil, leucovorin, irinotecan, and oxaliplatin) and nab-paclitaxel/gemcitabine, have emerged in the last decade as frontline standards of care, with response rates around 30–40%. Gemcitabine monotherapy is reserved for pts with lower performance status, substantial comorbidities, or other contraindications.

However, in each of these studies [3,4], the median overall survival of the pts remained less than one year, supporting the ongoing need to develop more beneficial therapies for this disease.

Pts who showed progression while receiving gemcitabine (+nab-paclitaxel) had a phase III-proven chance of further therapy, with a platinum- or irinotecan-based strategy combined with fluoropyrimidine infusion, if the performance status was sufficiently maintained [5,6,7]. After the first line with FOLFIRINOX, a strategy change to gemcitabine/nab-paclitaxel is possible but is rarely feasible and lacks any phase III-proven overall survival benefit.

In a small number of pts without significant vascular involvement (approximately 15–20%), radical cancer resection followed by adjuvant chemotherapy can offer a curative option [8,9,10]. Several classifications exist to define PDAC subgroups mainly by means of vascular contact or invasion. The National Cancer Centre (NCC) guidelines is the most commonly used to define the PDAC subtypes of resectable, borderline resectable (BRPC), and locally advanced pancreatic cancer (LAPC). While, in LAPC, primary resection is not recommended due to major venous thrombosis of the portal vein or superior mesenteric vein (SMV) or to circumferential encasement of the superior mesenteric artery (SMA), celiac axis, or proximal hepatic artery, BRPC, which is defined as the presence of tumor abutment on the portal vein or SMV and limited encasement of the mesenteric vein and portal vein, offers the option of a curative surgical resection [11]. However, in up to 50% of resected cancers, microscopic residuals are found in the resection margin. Consequently, the rate of local recurrence is high [12]. Preoperative therapy being mainly favored for BRPC increases R0 resection rates as a prognostic factor and for survival [13].

For pts with locally advanced inoperable PDAC but no evidence of distant metastasis, radio-chemotherapy is lacking effectiveness over chemotherapy alone [14]. However, with the use of a more effective systemic chemotherapy regimen developed in the last decade [3,4], the chance of response and furthermore of a secondary, curative-intended resection increases [15]. Resection rates for LAPC are described as up to 60% when treated with FOLFIRINOX [16], and the mOS can be significantly increased by secondary resection (35.3 vs. 16.3 months) [17]. This chance must be discussed in an interdisciplinary cancer conference of a high volume center at time of first diagnosis, especially since new operative methods such as celiac axis resection offer increased disease control with high rates of negative resection margins without increased perioperative mortality and complications [18,19]. As pancreatectomy with or without additional vessel resection remains a mayor surgical procedure with risks of complications, it is important to justify this procedure. In this context, it is the aim of current studies to identify prognostic factors that predict operability and the benefit of a secondary resection. While radiographic response alone has been proven to be misleading in terms of resectability [20], the tumor marker carbohydrate-antigen 19-9 (CA 19-9) might be a suitable and promising factor to support the selection of pts that benefit from this procedure [21].

The aim of this investigation was to identify factors that could predict or affirm the usefulness of a multimodal concept with a secondary resection after response to an induction treatment.

## 2. Materials and Methods

Pts undergoing systemic induction treatment followed by secondary resection for initially non-resectable PDAC in our cancer center in the period between March 2017 and May 2019 were identified from our Comprehensive Cancer Database. We collected the data prospectively. The analysis was done in a retrospective way. Inclusion criteria were the histologically proven presence of primarily non-resectable PDAC as well as the application of an undefined number of chemotherapy cycles before performed resection. We excluded other tumor-subtypes such as duodenal carcinoma, periampullary cancer, and neuroendocrine carcinoma. The PDAC was assessed as primarily non-resectable when anatomical signs for BRPC or LAPC were seen. The induction therapy was performed either inpatient or outpatient in our center or in an associated outpatient department. We discussed all pts upfront in our multidisciplinary cancer conference to assess for primary or secondary resectability and the suitability for appropriate chemotherapy treatment.

Data were analyzed for pre-, post-, and operative characteristics such as patient‘ characteristics including ECOG and Body Mass Index (BMI), therapeutic characteristics including the chosen neoadjuvant therapy, surgical procedures, side effects and complications, clinicopathological characteristics including CA 19-9 and Carcinoembryonic antigen (CEA) levels at diagnosis and after neoadjuvant treatment, and the pTNM status (histopathological state of tumor expansion, nodal state and metastasis) of the pts.

The classification and graduation of side effects during induction therapy were defined by Common Terminology Criteria for Adverse Events (CTCAE Version 5.0). Initial staging and restaging were performed via 3-phase CT scans. The radiographic response was defined by the RECIST criteria (1.1) (Response Evaluation Criteria in Solid Tumors) by varying specialists for radiology as follows: complete response (CR)—Disappearance of all target lesions; Partial Response (PR)—At least a 30% decrease in the sum of diameters of target lesions; Progressive Disease (PD)—At least a 20% increase in the sum of diameters of target lesions; Stable Disease (SD)—Neither sufficient shrinkage to qualify for PR nor sufficient increase to qualify for PD [22].

Statistics were performed using SPSS version 26 (IBM, New York, NY, USA). Fisher’s exact test, Chi-Square-test, *t*-test, and Wilcoxon’s test were used depending on scaling level. *p*-values were two-sided and considered to be statistically significant if *p* < 0.05. Kaplan–Meier curves were provided for survival estimations. We calculated survival from time of diagnosis to the death of the pt independent of the reason for death or to the last documented contact with the pt. The last group was defined as lost to follow up and was censored.

## 3. Results

### 3.1. Patient Characteristics

Forty pts (22 men and 18 women) fulfilled the inclusion criteria for analysis. The mean age at time of surgery was 61 years (37–82 years). Pretreatment parameters, such as ECOG status, American Society of Anesthesiologists (ASA) score, and BMI can be found in Table 1. The majority of the pts had an ECOG of 0 (66.7%) or 1 (30.3%) before initiation of chemotherapy. Only 2 pts (3%) showed an ECOG of 2. None of the pts had an ECOG below 2. Data for ASA score were captured before performance of the surgical resection and showed a majority for mild (ASA 2) and severe systemic diseases (ASA 3) in these pts (42.9% and 51.4%); 66.7% of the pts had a normal BMI (18.5–25 kg/m^2^), 29.6% were overweight (25.1–30 kg/m^2^), and 3.7% were underweight (<18.5 kg/m^2^). A 3-phase CT scan or surgical exploration offered reasons for primary irresectability.

### 3.2. Neoadjuvant Treatment

Most pts (57.7%) received induction therapy with FOLFIRINOX. One of them deescalated therapy to gemcitabine mono due to side effects after four applications. Seven pts (17.5%) received nab-paclitaxel and gemcitabine, whereas two external pts were treated with gemcitabine mono and cisplatin/capecitabine. In 8 pts (20%), a change in induction therapy between FOLFIRINOX and nab-paclitaxel/gemcitabine was performed. Four of the pts suffered from severe toxicity during induction therapy, which lead to a switch from FOLFIRINOX to nab-paclitaxel/gemcitabine. In one pt, the switch was administered because a significant increase in the tumor marker CA 19-9 was suspected to be progression of the disease. For two pts, a change from nab-paclitaxel/gemcitabine to FOLFIRINOX was planned within a clinical investigation.

### 3.3. Surgical Procedure

Depending on the localization of the tumor (Table 1) and the extent of infiltration, a surgical procedure was chosen. Performed surgeries were pylorus preventing or classical Whipple’s procedure and distal or total pancreatectomy (Table 2). Four pts underwent distal pancreatectomy with simultaneous resection of the celiac axis (Appleby procedure); 19 pts underwent an additional splenectomy; and 7 pts received a resection and reconstruction of the portal vein. Depending on the extent of local tumor infiltration, additional organ resections were performed (Table 2).

### 3.4. Side Effects and Complications

Of the pts, 51.6% suffered from side effects of grades 3 to 4 during preoperative therapy. Especially pts who received FOLFIRINOX and nab-paclitaxel/gemcitabine suffered from severe side effects (grades 3 to 4) significantly more often (*p* = 0.009). There was no difference in between the types of side effects depending on the performed induction therapy (Table 3). Of the pts, 56% sustained postoperative complications. Nine pts developed pancreatic fistulae. Five pts suffered from insufficiency of the biliodigestive anastomosis. One pt died from postoperative complications seven days after surgery due to a post pancreatectomy hemorrhage. The remaining seven pts with postoperative complications had either an impairment of wound healing or systemic inflammation and infection such as pneumonia. Resection of the portal vein or coeliac axis was not associated with a higher complication rate (*p* = 0.68; *p* = 0.60). There was also no difference in the rate of overall complications depending on the performed surgical procedure (*p* = 0.78).

### 3.5. Effect of Preoperative Therapy

Under induction therapy, the level of the tumor marker CA 19-9 changed from a mean of 4358.3 U/mL to 138.5 U/mL (*p* < 0.001), which is a decrease by 44.7%. The mean value of the tumor marker CEA dropped from 10.5 ng/L before induction therapy to 3.5 ng/L before surgery (*p* = 0.16). In 73% (27 of 37 pts) of the pts, CT scans showed partial response during induction therapy; 21.6% (8 of 37 pts) showed stable disease; and 5.4% (2 of 37 pts) showed progression of the disease. For three pts, there was no information available. The initial clinical tumor formula showed a cT4 stage in 54.1% (20 pts), a cT3 stage in 37.5% (14 pts), and a cT2 stage in 8.1% (2 pts). Histopathological analysis of the tumor showed a significant decrease of the T stage to pT4 in 15% (6 pts), to pT3 in 25% (10 pts), to pT2 in 27.5% (11 pts), and to pT1 in 20% (8 pts). In 5 pts (12.5%), there were no malignant cells detected at all (*p* = 0.000015) (Table 4). Meanwhile, the ECOG performance status of the pts worsened (*p* = 0.03) and the median BMI of the pts decreased slightly (*p* = 0.25) (Table 1). Ten pts (37%) suffered from weight loss of more than 5%.

### 3.6. Overall Survival

The median observation time was 19.5 months. The median OS from the time of diagnosis was 20 months (CI 95%: 17.2–22.9 months). The median OS from the time of resection was 17 months (CI 95%: 11.6–22.4). Nine patients (22.5%) were lost to follow up and censored.

### 3.7. Predictors of Overall Survival

**Figure 1:**
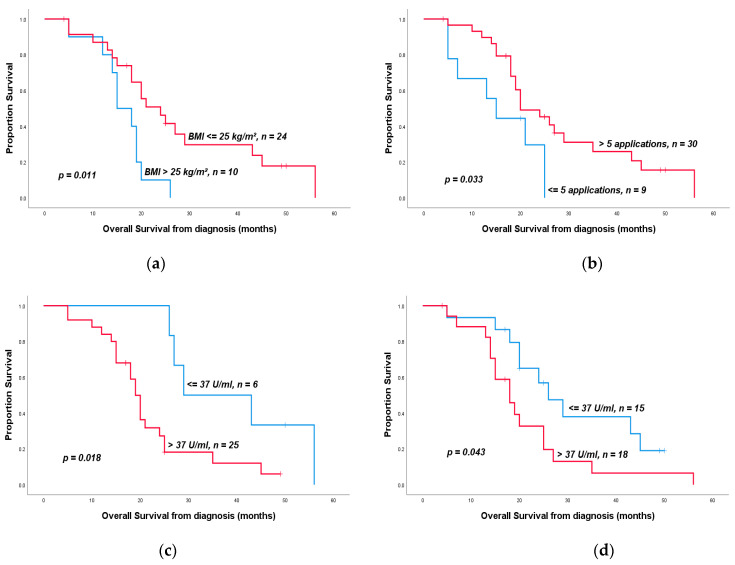
Kaplan–Meier overall survival curves in pts who underwent surgical resection after receiving neoadjuvant therapy: (**a**) overall survival depending on the body mass index (BMI) at the time after neoadjuvant therapy, (**b**) overall survival depending on the number of applications of neoadjuvant chemotherapy, (**c**) overall survival depending on the CA 19-9 level at the time of diagnosis, and (**d**) overall survival depending on the CA 19-9 level after neoadjuvant therapy.

Performing an adjuvant therapy (10 vs. 25 months, *p* < 0.001) as well as nodal negativity (25 vs. 15 months, *p* = 0.003) had positive impacts on the OS of the pts in our investigation. Pts who showed a normal weight or underweight after induction therapy (*p* = 0.01), pts who had a normal CA 19-9 levels (≤37 U/mL) at the time of diagnosis (*p* = 0.02) or after neoadjuvant therapy (*p* = 0.04) (Figure 1), and pts with a normalized CEA level (≤5 ng/L) after induction therapy profited from resection (*p* = 0.047). An elevated CA 19-9 level (>400 U/mL) after induction therapy tended towards significantly shorter OS (*p* < 0.001) (Table 5). The percentage of decrease in CA 19-9 level under induction therapy had no impact on the OS (exemplary decrease ≥ 75% vs. <75%, *p* = 0.41). Nevertheless, pts with a decrease in CA 19-9 levels over 90% were significantly more likely to receive R0 resection afterwards (*p* = 0.03). The age of the pts (≤60 vs. >60 years, *p* = 0.3), the pre- or post-therapeutic ECOG status (ECOG = 0 vs. >0, *p* = 0.75/0.6), the ASA score (ASA < 3 vs. ≥3, *p* = 0.5), the pretherapeutic BMI of the pts (≤25 vs. >25 kg/m^2^, *p* = 0.91), or the extent of weight loss during induction therapy (<5% vs. ≥5%, *p* = 0.45) had no impact on the OS. There was also no impact of the chosen induction therapy regimen (Table 5), whereas the number of applications of neoadjuvant chemotherapy had a significant influence on the OS (≤5 vs. >5 applications, *p* = 0.033) (Table 5, Figure 1). The total duration of neoadjuvant therapy had no impact on the OS of the patients (exemplary <3 months vs. ≤3 months, *p* = 0.89). There was no correlation between the number of applications and the presence of perineural (*p* = 0.16) or venous invasion (*p* = 1), or the pN (*p* = 0.71), R (*p* = 0.7), or pT stages (*p* = 0.69). Aside from the nodal status, the pT stage also had an impact on the OS. Pts with a pT stage of 0 or 1 showed significantly prolonged OS (43 vs. 19 months, *p* = 0.008) in comparison to higher T stages (Table 5). Although the absence of malignant cells in the histopathological resected part did not lead to a significantly prolonged OS (*p* = 0.22). A negative perineural infiltration had an impact on the OS, which was statistically not significant (*p* = 0.058) (Table 5). Further histopathological characteristics did not have an impact on the OS (pM 0 vs. 1, *p* = 0.67; R0 vs. ≥R1, *p* = 0.7; L0 vs. L1, *p* = 0.12; and V0 vs. V1, *p* = 0.33). The radiographic response measured by RECIST criteria (partial remission vs. stable/progressive disease, *p* = 0.82) as well as the presence of postoperative complications (*p* = 0.63) had no impact on the OS.

## 4. Discussion

Increasing the intended R0 resection rates is the main aim of preoperative treatment for initially non-resectable PDAC. When the option of resectability after initial treatment appears, it remains unclear which pts benefit from a secondary resection and if they reach a comparable OS to those with a primarily resected specimen [23] or rather suffer from early recurrence.

Our analysis showed an OS of up to 20 months for selected pts with initially non-resectable pancreatic cancer that mainly responded to induction chemotherapy. The mOS for pts with LAPC who did not undergo secondary resection is exemplarily described at 16.3 months [17]. We therefore believe that a secondary resection in pts after responding can be justified. However, the group of beneficiaries has to be narrowed down using prognostic or predictive factors.

Prognostic factors that are associated with an improved OS have been identified before, such as post-resectional nodal negativity [24]. Our results support this thesis once more as pts with nodal negativity had a significant longer OS compared to pts with nodal positivity. Preoperative treatment has a significant positive effect on nodal status [25]. Thus, we should establish a method to identify nodal positivity before secondary resection to discuss the benefit of an invasive surgery. An interpretation of the clinical nodal status via CT scan is difficult. Not every lymph node metastasis is suspicious in a CT scan and not every lymphadenopathy hides a metastasis especially after induction treatment when scar tissue may mimic viable tumor. In one study, only 54% of nodal statuses were interpreted correctly with the help of a CT scan [26]. Interpretation via PET-CT only reaches a sensitivity of 42% [27]. Finally, endosonographic biopsy of lymph nodes offers an almost hundred percent certainty about the histopathology of the tissue when an appropriate biopsy is succeeded [28]. Maybe pts would benefit from an upfront endosonographic biopsy of a suspicious lymphadenopathy to discuss further treatment in case of positivity.

Naumann et al. recently proved the impact of BMI on the outcome of pts treated with radiotherapy as induction [29]. Weight loss of more than 5% during induction therapy lead to a significant shorter overall survival (12 vs. 27 months). This result may outline weight loss as a symbol of high tumor activity, implying a missing benefit for secondary resection in these pts. On the contrary, our study showed a benefit for pts with normal weight and underweight compared to pts who are overweight. Obesity is associated with a higher risk for concomitant disease. Additionally, fat cells are identified as one cause for chronic inflammation [30]. Earlier works already showed the negative impact of obesity on the outcome of pts with pancreatic cancer [31]. Therefore, further studies should examine the role of obesity or even cachexia on the outcome of pts with PDAC.

Our study also revealed a benefit of secondary resection for pts with normal pretherapeutic CA 19-9 levels. These types of cancer may have a less aggressive spreading biology than those with elevated CA 19-9. A previous research showed that the tumor marker CA 19-9 itself promotes the activation of EGFR (Epidermal Growth Factor Receptor) signaling in mice, suggesting an important role in the initiation and acceleration of pancreatic cancer [32]. Additionally, Gao et al. demonstrated the role of the gene FUT 3 (Galactoside 3(4)-L-fucosyltransferase) or the Lewis gene that is responsible for fucosylation of proteins and the synthesis of CA 19-9. Fucosylation by FUT 3 was shown to be upregulated in metastatic PDAC, suggesting a relation to the promotion of motility in pancreatic cancer [33]. Five to ten percent of the individuals were homozygous for the recessive allele of the gene FUT 3. These pts showed lower levels of fucosylation and thus no or low CA 19-9 secretion [34]. This leads to the supposition that pancreatic cancer in Lewis-negative pts should have a less aggressive biology. A previous study showed the opposite. In Liu et al., Lewis-negative pts with pancreatic cancer had a significant shorter overall survival and showed a higher proportion of metastasis. Possible reasons for this phenomenon could be the upregulation of CA 125 in these cases acting as a promotor for tumor cells or the lack of fucosylation playing an important role in human body physiology [34].

Furthermore, supporting the role of CA 19-9, the pts in our study who had an elevated CA 19-9 level >400 U/mL after induction therapy did not profit from resection. A normalized CA 19-9 level through induction therapy even lead to a significantly better OS for the pts, suggesting the tumor marker as a convenient predictive factor. The extent of the decrease in CA 19-9 did not have an impact on overall survival but on resection rate quality. If there was a decrease by more than 90%, these pts showed a higher rate of R0 resections. Similar results were seen in Boone et al., where a decrease of more than 50% was associated with a higher R0 resection rate in borderline pancreatic cancer [35].

Another impact on the OS of pts was the number of applications of neoadjuvant chemotherapy. Pts who were able to receive at least 5 applications benefited more from a secondary resection. Possible reasons could be the reduction of perineural or venous infiltration by prolonged chemotherapy, although our analysis did not show significant results to support this thesis. A limitation of this finding might be the unsuitability of the unit “applications” instead of “cycles” as one cycle consists of a different number of applications. Nevertheless, based on this result, we should consider finding-specific ranges for the duration of neoadjuvant therapy. Few studies exist that have investigated this question. While Truty et al. showed a significant benefit for pts who received at least 6 cycles of FOLFIRINOX or gemcitabine/nab-paclitaxel [36], another study proposed 4 cycles of modified FOLFIRINOX as sufficient for neoadjuvant therapy in BRPC [37]. Further investigations are necessary to establish a recommendation.

The second interest of this study was to identify factors which predict the ability of resection after induction treatment. CA 19-9 was one of the promising factors, although several studies revealed heterogeneous results. Michelakos et al. demonstrated the role of CA 19-9 in BRPC by showing that pts who were resected after induction therapy had a lower CA 19-9 level after induction than pts who could not be resected (21 vs. 40 U/mL) although CA 19-9 level was normalized in almost all of the pts [38]. The pts in our study had a mean CA 19-9 level of 138.5 U/mL after induction therapy, which is still higher than that in those not resected in the study of Michelakos et al. Therefore, we cannot support the approach of using specific strong threshold values of CA 19-9 by itself for decision making in PDAC.

The rate of radiographically measured by partial remission under induction therapy was particularly high in this study, at 73%. Compared with other studies where partial response under induction therapy with FOLFIRINOX reached 31% [3] and under gemcitabine/nab-paclitaxel reached in 23% [4], radiographic response offers itself as a promising factor. Oppositely, studies have shown that RECIST criteria being used in order to interpret response under induction therapy with FOLFIRINOX are diagnostically less conclusive. Katz et al. demonstrated that 66% of their pts with borderline resectable pancreatic cancer were resected after induction therapy although CT scans showed non-resectable situations [20]. The borders of CT imaging for PDAC treated with neoadjuvant therapy lie in the disability of distinguishing between viable cancer and fibrosis as it occurs after neoadjuvant treatment. Therefore, specialists suggest exploring all pts independently of radiographic results and deciding on resection depending on frozen-section biopsies of involved arteries and venous structures [39].

Finally, to evaluate the reasons for worthwhile resectability in our cohort, it is necessary to combine multiple criteria. Many of those are already essential components for decision making within multidisciplinary tumor conferences. To be suitable for an intensive neoadjuvant therapy such as FOLFIRINOX, pts need to have an ECOG level of at least 1, which fulfilled 96% of our pts. Even after induction treatment, 81.4% had an ECOG of at least 1, suggesting a good tolerance for further multimodal treatments. Secondly, our pts showed a significant decrease in the tumor marker CA 19-9 from a mean of 4358.3 U/mL to 138.5 U/mL. Even though the degree of decline of CA 19-9 after neoadjuvant therapy had no significant influence on the survival of the pts, this sharp decline in the tumor marker in combination with the high number of radiographic response could have been seen as predictive for the success of a secondary resection.

The present study is limited by common biases that are mainly due to the retrospective character of this analysis. Pts that are able to undergo secondary resection initially profited from the selected chemotherapy and might also profit from continuing with this therapy regime. Prospective randomized trials are necessary to evaluate the benefit of a secondary resection over a continued chemotherapy in these cases. However, ongoing chemotherapy in cases of initial remission might eventually lead to higher rates of side effects due to toxic bone marrow damage and resistances against the chosen chemotherapy, which furthermore supports the role of a definite resection. Another limitation of this investigation is the small number of pts as well as the missing differentiation between LAPC and BRPC. However, we need to discuss whether this classification becomes obsolete in this new era of neoadjuvant therapy when LAPC becomes resectable and, in some cases, even reaches comparable OS to initially resectable pancreatic cancer [23].

Furthermore, interpretation of the radiographic response and clinical T status of locally advanced PDAC is limited. As already described above, CT scans have limitations in distinguishing viable cancer tissue and fibrosis. Additionally, a review of the American National Cancer Data Base showed a poor correlation between the clinical and pathological stages in pts who received initial surgery [40]. Consequently, results regarding clinical stage should be considered with caution.

## 5. Conclusions

Pancreatic cancer remains a disease with a generally poor outcome but heterogeneous clinical course. To identify the small but important number of pts who could potentially benefit from individual strategies, interdisciplinary, experienced cancer boards must involve the following factors in decision-making: the radiographic response, the course of tumor markers, and the general performance status of the pts. The initial CA 19-9 level does not seem to be suitable to predict the probability of a secondary resection; however, it provides the opportunity to predict the chance of a resection and prolonged survival during the course and should influence decision-making.

Nodal status plays an important role in the OS of pts, suggesting a non-localized disease. Special attention should be paid to the diagnosis of nodal positivity after induction treatment either by endosonographic biopsies of suspicious lymphadenopathies or by explorative surgeries with frozen-section analyses of lymph nodes.

## Figures and Tables

**Table 1:** Patient characteristics.

Baseline Characteristics (*n* = 40)					
**Age**					
Mean	60.6 (37–82) years				
**Gender**					
Male	22 (55.0%) pts				
Female	18 (45.0%) pts				
**Primary Localization**					
Head	21 (52.5%) pts				
Korpus	13 (32.5%) pts				
Tail	6 (15%) pts				
	**At Diagnosis**		**After Induction/Before Resection**		
	***N***	**%**	***N***	**%**	***p***
**ECOG**					0.03
0	22	66.7	10	37	
1	10	30.3	12	44.4	
2	1	3	5	18.5	
n.a.	7		13		
**ASA Score**					
1			2	5.7	
2			15	42.9	
3			18	51.4	
n.a.			5		
**BMI [kg/m^2^]**	Median 23.9 (17.2–28.7)		Median 22.6 (16.8–29.9)		
<18.5	1	3.7	1	2.9	0.25
18.5–24.9	18	66.7	23	67.6	
≥25	8	29.6	10	29.4	
n.a.	13		6		

n.a.—not available, BMI—body mass Index, ASA—American Society of Anesthesiologists, and ECOG—Eastern Cooperative Oncology Group.

**Table 2:** Therapeutic characteristics.

Parameter	Category	*N*	%
Induction Therapy			
	FOLFIRINOX	23	57.5
	Nab-Paclitaxel/Gemcitabine	7	17.5
	FOLFIRINOX ► Nab-Pac/Gem	8	20
	Gemcitabine mono	1	2.5
	Cisplatin/Capecitabine	1	2.5
Number of Applications			
	Median	8 (3–25)	
	≥8 Applications	20	51.3
	<8 Applications	19	48.7
	n.a.	1	
Duration of neoadjuvant Therapy			
	Median (months)	3 (1–24)	
	>3 months	18	48.6
	≤3 months	19	51.4
	n.a.	3	
Adjuvant Therapy			
	Yes	15	75
	No	5	25
	n.a.	20	
Surgical Procedure			
	PPPD	16	40
	Whipple’s Procedure	2	5
	Distal pancreatectomy	13	32.5
	Total pancreatectomy	9	22.5
Additional resection			
	Splenectomy	19	47.5
	Gastrectomy (total/partial)	2	5
	Partial hepatectomy	3	7.5
	Resection of portal vein	7	17.5
	Resection coeliac axis	4	10
	Hemicolectomy	1	2.5
Post Op-Complications			
	BDA-Insufficiency	26	65
	Pancreatic fistula	5	12.5
	Postpancreatectomy	9	22.5
	haemorrhages	1	2.5

n.a.—not available, PPPD—Pylorus-Preserving Pancreaticoduodenectomy, and BDA—Biliodigestive Anastomosis.

**Table 3:** Side effects during induction therapy.

		Total		Folfirinox		Nab-Paclitaxel/Gem		Folfirinox ►Nab-Pac/Gem		*p*
Parameter	Category	*N*	%	*N*	%	*N*	%	*N*	%	
General Side effects	0–2	15	48.5	19	63.2	5	60	7	0	0.009
				12		3		0		
	3–4	16	51.6	7	36.8	2	40	7	100	
Neutropenia				15		5		7		
	0–2	16	59.3	11	73.3	3	60	2	28.6	0.16
	3–4	11	40.7	4	26.7	2	40	5	71.4	
Anemia				17		5		8		
	0–2	26	86.7	15	88.2	4	80	7	87.5	1
	3–4	4	13.3	2	11.8	1	20	1	12.5	
Thrombopenia				9		3		4		
	0–2	15	93.8	8	88.9	3	100	4	100	1
	3–4	1	6.3	1	11.1	0	0	0	0	
Infections				16		4		7		
	0–2	24	88.9	15	93.8	4	100	5	71.4	0.2
	3–4	3	7.5	1	6.2	0	0	2	28.6	
Diarrhea				4		1		3		
	0–2	5	62.5	3	75	1	100	1	33.3	0.68
	3–4	3	37.5	1	25	0	0	2	66.7	
Nausea				6		3		6		
	0–2	15	93.8	6	100	2	66.7	5	83.3	0.66
	3–4	2	13.3	0	0	1	33.3	1	16.7	
Neurological side effects	0–2	13	81.3	10	70	3	100	3	100	0.52
				7		3		3		
	3–4	3	18.8	3	30	0	0	0	0	

**Table 4:** Clinical-pathological characteristics.

		At Diagnosis				After Induction Therapy				
Parameter	Category	*N*	%	Mean	IQR	*N*	%	Mean	IQR	*p*
CA 19-9 (U/mL)				4358.3	64.8–830			138.5	12.1–65.4	0.001
	≤37 U/mL	5	16.1			15	44.1			
	37–400 U/mL	14	45.2			17	50			
	>400 U/mL	12	38.7			2	5.9			
	n.a.	9				6				
CA 19-9 trend (-%)		29		44.7	18–94.75					
CEA (ng/mL)				10.5	2.5–7			3.5	2–5.3	0.16
	≤5 ng/ml	15	65.2			18	69.2			
	>5 ng/ml	8	34.8			8	30.8			
	n.a.	17				14				
c/pT Status										0.001
	0	0	0			5	12.5			
	1	0	0			8	20			
	2	3	8.1			11	27.5			
	3	14	37.8			10	25			
	4	20	54.1			6	15			
	n.a.	3				0				
pN Status										
	0					21	52.5			
	1					19	47.5			
c/pM Status										0.18
	0	30	78.9			35	87.5			
	1	8	21.6			5	12.5			
	n.a.	2				0				
R Status										
	0					18	46.2			
	1					21	53.8			
	n.a.					1				

n.a.—not available, c/pT—clinical/histopathological tumor expansion, pN—histopathological nodal status, c/pM—clinical/histopathological distant metastasis, R—residual tumor, CA 19-9—carbohydrate-antigen 19-9, and CEA—Carcinoembryonic antigen.

**Table 5:** Impact factors on overall survival.

Parameter	Category	OS (Months)	*p*	HR	95%CI	*p*
CA 19-9 at diagnosis	≤37 vs. >37 U/ml	29 vs. 19	0.02	3.44	1.14–10.36	0.028
CA 19-9 after induction	≤37 vs. >37 U/mL	26 vs. 18	0.043	2.23	0.99–5.03	0.054
	<400 vs. ≥400 U/mL	24 vs. 7	0.001	17.2	2.38–124.45	0.005
CEA after induction	≤5 vs. >5 ng/ml	25 vs. 18	0.047	2.67	0.96–7.48	0.061
BMI after induction	>25 vs. ≤25	15 vs. 24	0.01	0.36	0.15–0.83	0.017
pN status	0 vs. 1	25 vs. 15	0.003	2.99	1.39–6.41	0.005
pT status	0–1 vs. 2–4	43 vs. 19	0.008	3.09	1.26–7.62	0.014
Pn	0 vs. 1	35 vs. 19	0.058	2.46	0.92–6.56	0.072
Neoadjuvant protocoll	FOLFIRINOX vs. other	20 vs. 20	0.88	0.95	0.46–1.97	0.89
	Nab-Pac/Gem vs. other	20 vs. 20	0.54	0.77	0.33–1.81	0.55
	FOLFIRINOX ► Nab-Pac/Gem vs. other	18 vs. 20	0.22	1.86	0.68–5.11	0.23
Number of neoadjuvant applications	≤5 vs. >5	15 vs. 20	0.033	0.41	0.17–0.98	0.044
Adjuvant therapy	no adjuvant therapy vs. adjuvant therapy	10 vs. 25	0.001	0.21	0.08–0.55	0.002

CA 19-9—carbohydrate-antigen 19-9, CEA—Carcinoembryonic antigen, BMI—body mass index, pN—histopathological nodal status, pT—histopathological tumor expansion, and Pn—perineural invasion.

## Data Availability

The data presented in this study are available on request from the corresponding author. The data are not publicly available due to private property of Charité–Universitätsmedizin Berlin.
